# Methylation Markers for Early Detection and Differentiation of Follicular Thyroid Cancer Subtypes

**DOI:** 10.5539/cco.v4n2p1

**Published:** 2015-06-15

**Authors:** Josena K. Stephen, Kang Mei Chen, Jason Merritt, Dhananjay Chitale, George Divine, Maria J. Worsham

**Affiliations:** 1 Department of Otolaryngology/Head and Neck Research, Henry Ford Hospital, Detroit, Michigan 48202, USA; 2 Department of Pathology, Henry Ford Hospital, Detroit, Michigan 48202, USA; 3 Department of Public Health Sciences, Henry Ford Hospital, Detroit, Michigan 48202USA

**Keywords:** follicular thyroid cancer, DNA methylation, QMSP, Hurthle cell cancer

## Abstract

Thyroid cancer has the fastest rising incidence rates and is the fifth most common cancer in women. There are four main types of which the papillary and follicular types together account for >90%, followed by medullary cancers (3%−5%) and anaplastic carcinomas (<3%). For individuals who present with early stage disease of papillary and follicular cancers, there are no accurate markers to predict whether they will develop metastatic or recurrent disease. Our immediate goal is to molecularly differentiate follicular cancer subtypes for enhanced classification. Promoter methylation status of genes with reported associations in thyroid cancer (*CASP8, CDKN2A, DAPK1, ESR1, NIS, RASSF1* and *TIMP3*) were examined in a cohort of follicular thyroid cancers comprising of 26 Hurthle and 27 Classic subtypes utilizing quantitative methylation-specific PCR. *RASSF1* was differentially methylated in Classic tumor tissue compared to Hurthle (p<0.001). Methylation of *RASSF1* pointed to racial group differences between African Americans and Caucasian Americans (p=0.05). Extra thyroidal extension was found to be associated with *DAPK1* (p=0.014) and *ESR1* (p=0.036) methylation. Late stage disease was associated with older age (p<0.001) and methylation of *DAPK1* (p=0.034) and *ESR1* (p=0.035). The methylation status of *RASSF1, DAPK1* and *ESR1* suggests the utility of methylation markers to molecularly differentiate thyroid cancer subtypes for enhanced classification and early detection of thyroid cancer.

## 1. Introduction

Thyroid cancer (TC) is the most common endocrine malignancy, accounting for 95% of all endocrine malignancies (Boufraqech, Patel, Xiong, & Kebebew, 2013), and which has an increasing incidence for 1999-2008: going up by an estimated 6.2% per year for men and 7.3% per year for women (Simard, Ward, Siegel, & Jemal, 2012). Unfortunately, neoplasias of the thyroid have been largely ignored because of the overall favorable prognosis of papillary thyroid cancers (PTC) and follicular thyroid cancers (FTC), with 5-year survival rates of approximately 98% when submitted to timely and appropriate treatment (SEER Cancer Statistics Factsheets: Thyroid Cancer. 2014). Despite this favorable prognosis, the increasing incidence is of concern. Moreover, no significant progress has been made to improve survival. Those diagnosed at late stages have a devastating 5-year survival rate of under 60% (Weber & Eng, 2005). Also, the recurrence rate of thyroid cancer is high (30%) (Mazzaferri & Kloos, 2001), and only one third of patients with distant metastases respond to radio-iodine (^131^I) therapy with complete remission (Schlumberger, 1998). In addition, there is a lack of accurate preoperative markers or molecular-based predictive models to differentiate benign nodules (follicular adenomas [FA]) from follicular cancer. Thus, despite advances on scientific and clinical fronts, many advanced thyroid cancers remain incurable.

The majority of thyroid nodules are benign so their clinical importance is related to excluding thyroid cancer. Fine-needle aspiration (FNA) biopsy is the standard diagnostic tool for preoperative diagnosis (Segev, Clark, Zeiger, & Umbricht, 2003). However, it has some limitations, especially in the presence of follicular lesions. A single false-negative FNA can delay surgical treatment by 28 months even with clinical evidence suggesting malignancy. This may lead to higher rates of vascular and capsular invasion, persistent disease at follow up, and advanced stage disease making it incurable (Yeh, Demircan, Ituarte, & Clark, 2004). For individuals who present with early stage disease of either PTC or FTC, there is no accurate marker(s) to predict whether they will develop metastatic or recurrent disease.

New approaches are necessary to identify potential novel diagnostic and prognostic markers, which would allow for more accurate early diagnoses, with personalized clinical management and surveillance. The present challenge is to obtain reliable preoperative markers that differentiate benign and malignant thyroid nodules, to detect early thyroid cancer, to improve discrimination of adenoma, PTC, and FTC so as to delineate thyroid subtypes in order to improve patient management. In attempting to tackle these challenges, this study’s goal was to identify DNA methylation markers that molecularly differentiate FTC subtypes, specifically FTC-Classic and FTC-Hurthle, for enhanced classification which can eventually lead to early detection of FTC.

## 2. Method

### 2.1 Cohort

This retrospective study cohort of 53 follicular thyroid cancers (26 FTC-Hurthle and 27 FTC-Classic subtypes) comprised of 14 males and 39 females ranging in age from 18 to 86 years with a race distribution of 34 Caucasian American (CA), 14 African American (AA), and 5 unknown race (Table 1).

The cohort was drawn from a large, clinically well-characterized multiethnic (33% AA) primary care patient population in the Detroit, Michigan area. Patients 18 years or older diagnosed with a primary thyroid cancer in the Henry Ford Health System between 1983 and 2012 with available tumor tissue blocks were identified through tumor registry records. All tumors were staged using the American Joint Committee on Cancer (AJCC) TNM system (American Joint Committee on Cancer, 2010). Cases staged as 1, 2 and unknown stage (2 cases) were grouped as Early stage while stages 3 and 4 were grouped together as Late stage disease. Risk factor information of race (as self reported), age, gender, and marital status was obtained through medical record abstraction. Promoter methylation status of *CASP8, CDKN2A, DAPK1, ESR1, NIS, RASSF1* and *TIMP3*, genes with reported associations in thyroid cancer, were examined utilizing quantitative methylation-specific PCR (QMSP). This study was approved by the Henry Ford Health System Institutional Review Board committee.

### 2.2 DNA Extraction

Whole 5 micron tissue sections or microdissected thyroid lesions and adjacent normal when present were processed for DNA extraction as previously described (Chen, Sawhney, Khan, Benninger, & Hou, 2007).

### 2.3 Bisulfite Modification and Quantitative Methylation-Specific Polymerase Chain Reaction (QMSP) Assay

Genomic DNA (~100 ng) from formalin-fixed paraffin embedded thyroid tissue and control universal methylated DNA (Chamicon International, Inc) were modified using the EZ-96 DNA Methylation Lightning Kit (Zymo Research, Orange, CA) during which methylated DNA is protected and unmethylated cytosine is converted to uracil (Chen et al., 2007).

QMSP detects the presence of tumor-specific DNA with a sensitivity of 1 cell in 1000 normal cells (Harden, Tokumaru, Westra, Goodman, & Ahrendt, 2003). This PCR approach is more sensitive than conventional PCR and more specific due to the use of an internally binding, fluorogenic hybridization probe (Eads, Danenberg, Kawakami, Saltz, & Blake, 2000; Lo, Wong, Zhang, Tein, & Ng, 1999). An advantage of QMSP is that it can measure the amount of methylation in a sample. Primers and probes (Table 2) were specifically designed to amplify *CASP8, CDKN2A, DAPK1, ESR1, NIS, RASSF1* and *TIMP3* genes. Primers and probes to an internal reference gene (*ACTB*) were run in parallel to standardize the input DNA.

PCR was carried out using the EpiTect MethyLight PCR Kit (Qiagen, Valencia, CA) according to the manufacturer’s protocol in 384-well plate using the 7900HT Sequence detector (Applied Biosystems). Each plate included multiple water blanks and serial dilutions of a universal methylated positive control DNA (100ng/μl, Chemicon, Temecula, CA) which was bisulfite converted in the lab for constructing the standard curve. These were run with the samples on each plate in duplicate. To determine the relative levels of methylated promoter DNA in each sample, the values of the gene of interest were compared with the values of the internal reference gene (*ACTB*) to obtain a ratio that is then multiplied by 100 to give a percentage value (gene of interest/reference gene X 100). The methylation results obtained by QMSP in this cohort are considered as a binary event in which any quantity of methylation in a sample is considered as positive (> 0.0) (Carvalho, Henrique, Jeronimo, Nayak, & Reddy, 2011).

### 2.4 Statistical Analysis

Comparisons were made between AA and CA, early and late stage disease and between patients with and without extra thyroidal extension findings, by t-test (for age), chi square tests (for categorical variables), and by Wilcoxon rank sum tests (for QMSP values). The distributions of QMSP values for the two groups were displayed using dot plots. In a few instances of patients who did not have values available for race, stage or extra thyroidal extension, comparisons were performed using all usable data for each comparison and the sample sizes available for each analysis were noted and reported.

## 3. Results

**Figure 1:**
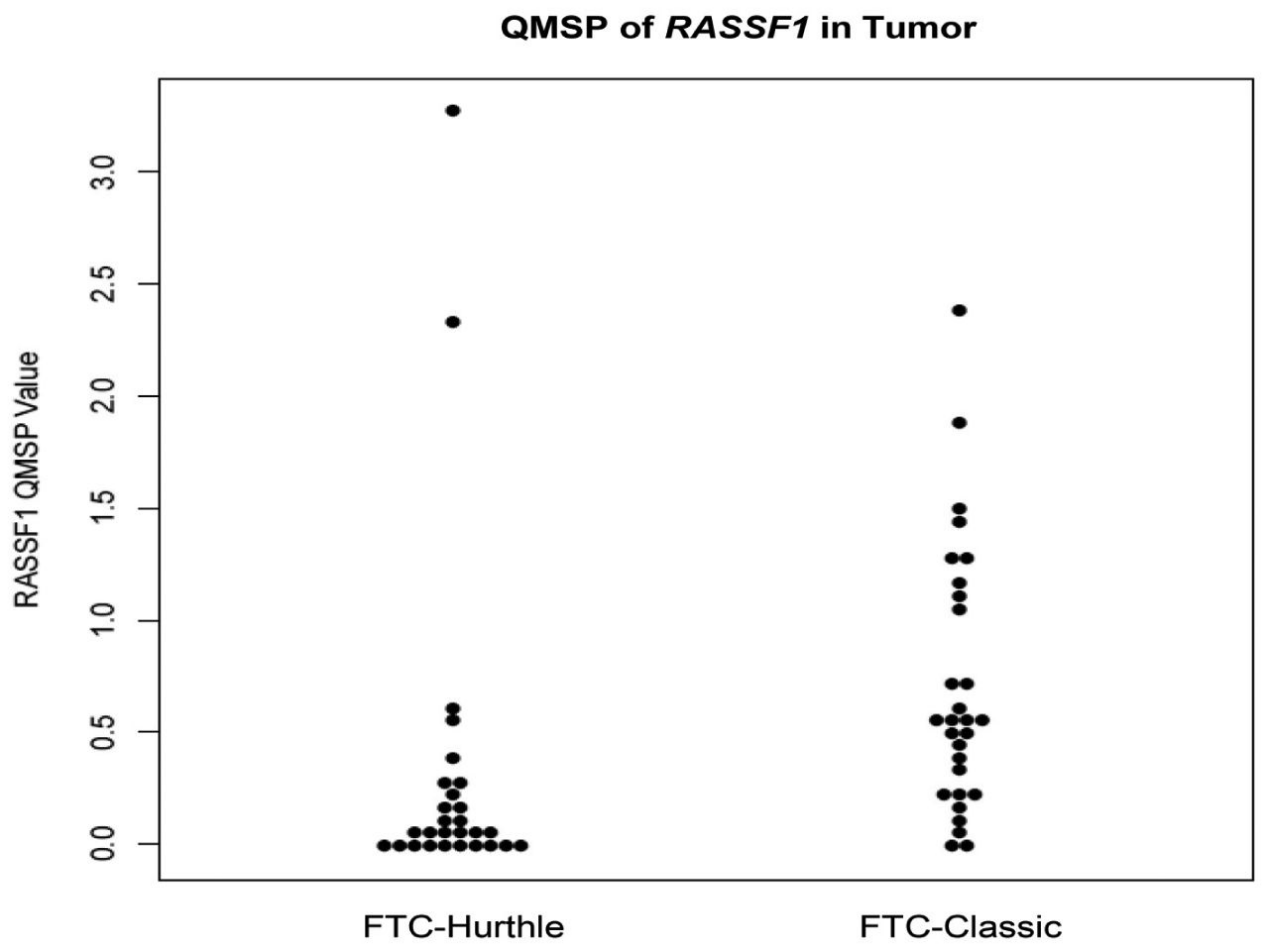
QMSP of *RASSF1* in Tumor (FTC-Hurthle vs FTC-Classic): Dot plot chart of QMSP values for FTC-Hurthle and FTC-Classic cases demonstrating differences in methylation levels of *RASSF1* (p<0.001) between the two tumor groups. (FTC - follicular thyroid cancer)

**Figure 2:**
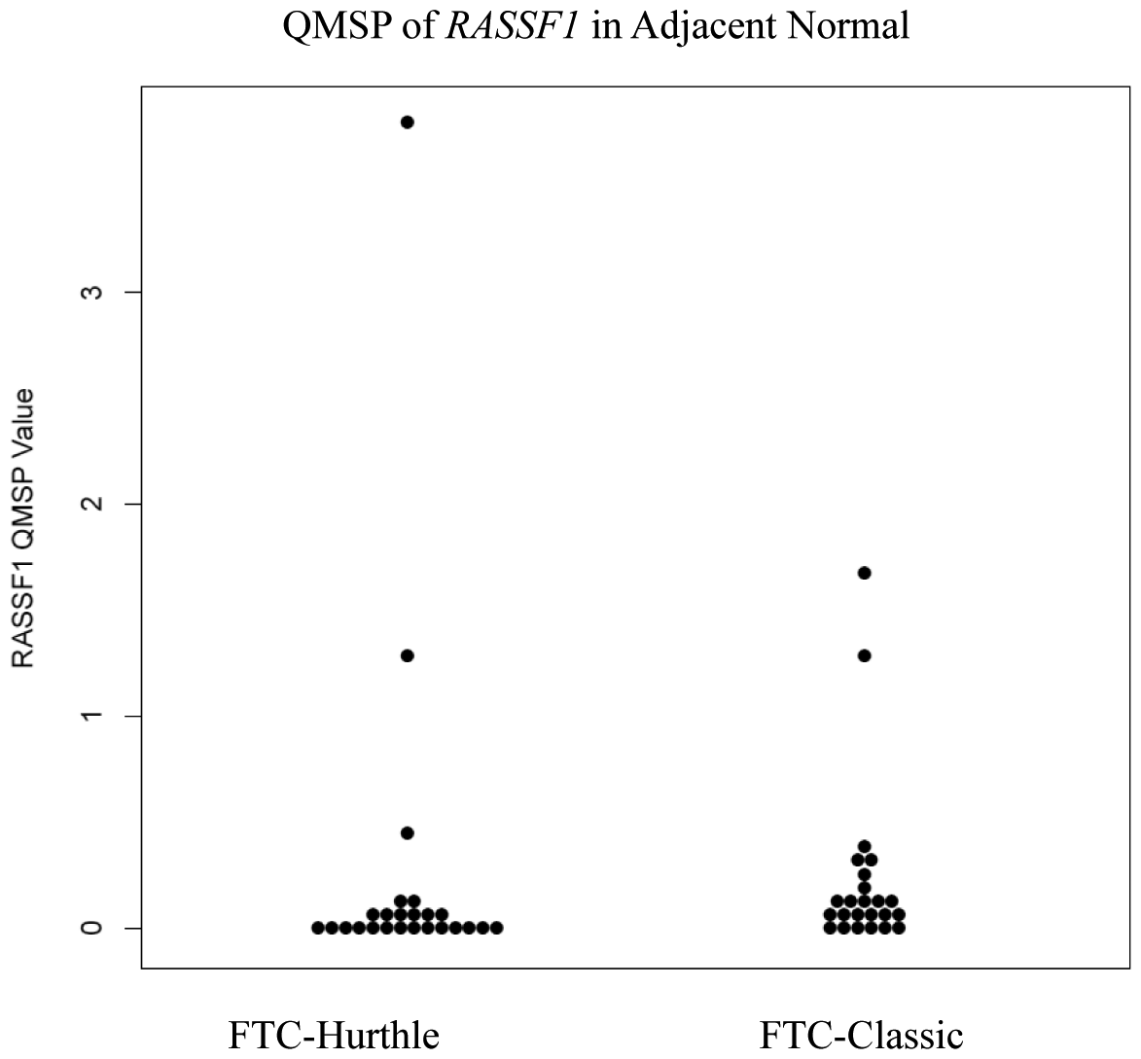
QMSP of *RASSF1* in Adjacent Normal (FTC-Hurthle vs FTC-Classic): Dot plot chart of QMSP values for FTC-Hurthle and FTC-Classic cases demonstrating differences in methylation levels of *RASSF1* (p=0.01) between the adjacent normal lesions for the two groups. (FTC - follicular thyroid cancer)

**Figure 3:**
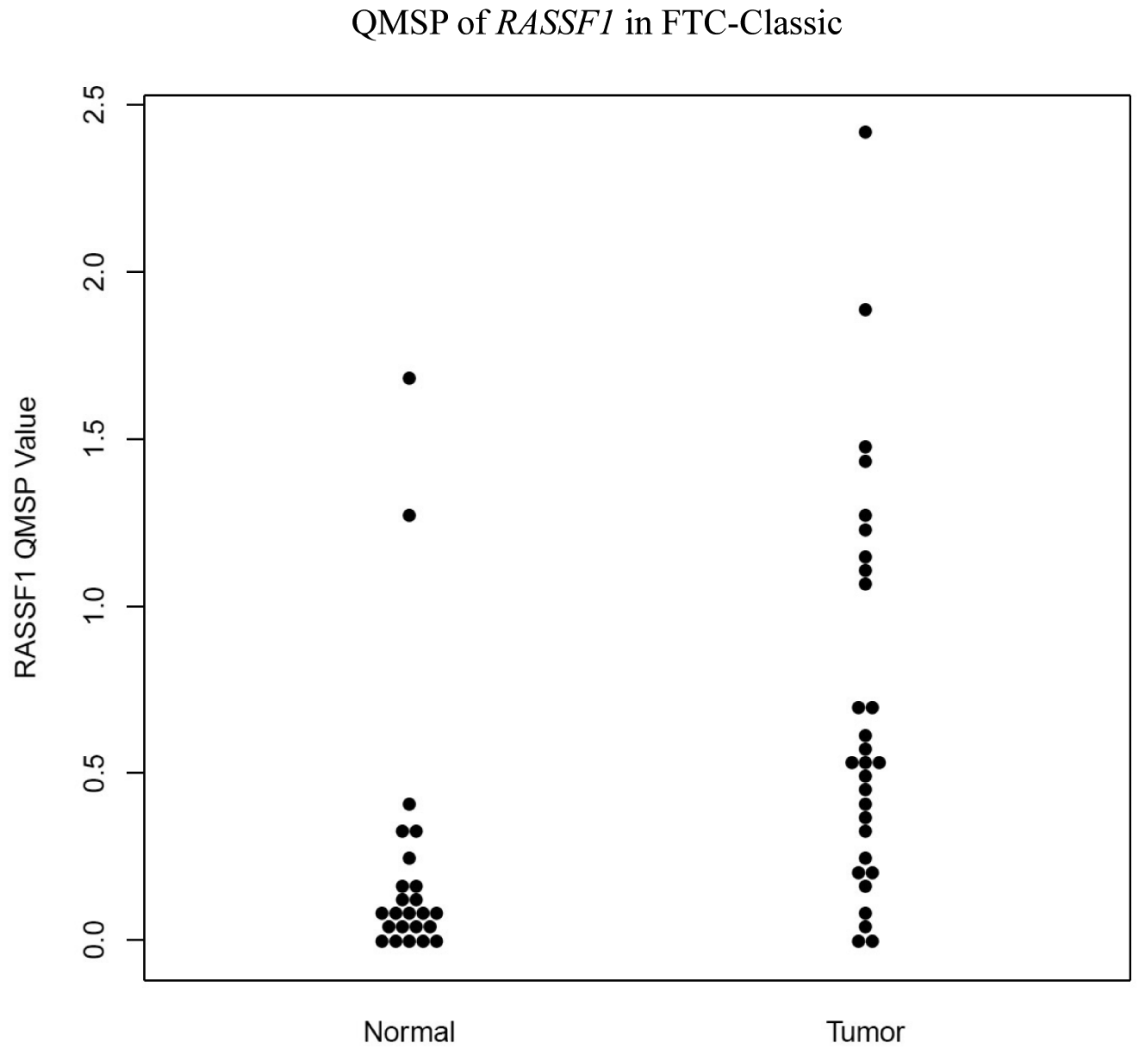
QMSP of *RASSF1* in FTC-Classic (Tumor vs Adjacent Normal): Dot plot chart of QMSP values for FTC-Classic cases demonstrating differences in methylation levels of *RASSF1* (p <0.001) between the adjacent normal and tumor lesions. (FTC - follicular thyroid cancer)

The retrospective cohort comprised of 26 FTC-Hurthle and 27 FTC-Classic thyroid cancer subtypes. Comparisons between FTC-Classic and FTC-Hurthle tumor tissue demonstrated significant differences in *RASSF1* methylation levels (p<0.001, Table 3, Figure 1) with higher levels in the FTC-Classic tumor tissue. Comparisons between their adjacent normal tissues were also significant (p=0.01). Within each FTC subtype, the tumor tissue demonstrated higher methylation levels compared to the adjacent normal, but was significant for the FTC-Classic subtype (p<0.001, Table 4, Figures 2 and 3). *DAPK1*, *ESR1, NIS* and *TIMP3* demonstrated methylation (QMSP values >0.0) but their levels were not statistically significant between subtypes. *CASP8* and *CDKN2A* were not methylated in any samples (all QMSP values were 0). Comparisons between AA and CA, early and late stage disease and between patients with and without extra thyroidal extension findings for age, gender, other categorical variables (angiolymphatic invasion and capsular invasion), and QMSP values were performed (Table 3). *RASSF1* methylation levels pointed to race group differences with higher mean methylation values in AA than CA (0.637 vs 0.422, p=0.05). Extra thyroidal extension is associated with *DAPK1* (p=0.014) and *ESR1* (p=0.036) methylation. Late stage disease is associated with older age (p<0.001, average age 66.7 years) and methylation of *DAPK1* (p=0.034) and *ESR1* (p=0.035). Angiolymphatic invasion, capsular invasion and gender (data not shown) did not show any significant association with gene methylation, age, race, stage or extra thyroidal extension.

## 4. Discussion

Early detection of cancer before metastasis is important for patients and clinicians as it can improve prognosis, patient quality of life and provide additional treatment options. One of the best ways for early detection of cancer is through the use of biomarkers. Several immunohistochemical and molecular markers for thyroid tumorigenesis have been proposed and some have been validated on a large scale for use in routine practice.

Various molecular alterations (mutations and/or gene rearrangements) have been described in the development of thyroid cancers. Genetic rearrangements which result in activation of the *RET* proto oncogene was the first recognized molecular event found in PTC especially in those exposed to ionizing radiation (Eberhardt, 2003). Chromosomal rearrangement resulting in a fusion gene, *PAX8/PPAR*γ, may be involved in follicular adenoma (FA) to FTC progression (Eberhardt, 2003). Currently, several types of gene mutation and expression panels are being tested for introduction into clinical practice. Mutational markers for FNA biopsy include *BRAF* and *RAS* point mutations and *RET/PTC* and *PAX8/PPARg* rearrangements, and possible *TRK* rearrangement (Nikiforov, Ohori, Hodak, Carty, & LeBeau, 2011; Nikiforov, Steward, Robinson-Smith, Haugen, & Klopper, 2009; Cantara, Capezzone, Marchisotta, Capuano, & Busonero, 2010). A limitation of genetic testing for molecular alterations is the low incidence of mutations, resulting in low sensitivity for diagnosing thyroid cancer in indeterminate nodules (Patel, Goyal, & Goldenberg, 2014). The Afirma Gene Expression Classifier, an mRNA based test utilizing FNA, analyzes the expression of 142 genes (167 RNA transcripts) using a proprietary algorithm to assign indeterminate thyroid nodules into either benign or suspicious groups (Chudova, Wilde, Wang, Wang, & Rabbee, 2010) and is currently commercially available. However, it is costly and does not discriminate amongst various thyroid lesions. A major limitation of the Afirma test is its low specificity (52%) in indeterminate nodules (Patel et al., 2014). The main concern for many endocrinologists is that it misclassifies lesions as suspicious about half the time.

Epigenetic alterations in the form of aberrant DNA methylation of tumor suppressor genes have been reported in many cancers. Methylation of *DAPK1* and *RASSF1* genes is known to strongly contribute to carcinogenesis, metastasis and treatment failure in various types of cancer (Wong, Chang, Tang, Wei, & Kwong, 2002; Supic, Kozomara, Brankovic-Magic, Jovic, & Magic, 2009). Hypermethylation of *RASSF1*, a known tumor suppressor gene, has been described in 75% of FTC, a smaller percentage of benign adenomas (44%), and PTC (20%) (Xing, Cohen, Mambo, Tallini, & Udelsman, 2004) indicating that this may be an early step in follicular cell derived thyroid tumorigenesis. In a pilot study of 21 thyroid cases by our group (Stephen, Chitale, Narra, Chen, & Sawhney, 2011), *RASSF1, CASP8* and *NIS* were frequently methylated in normal thyroid samples, hyperthyroid nodules and in thyroid cancer cases (matched for presence of normal and tumor tissue from the same biopsy specimen), suggesting these as early changes in thyroid tumorigenesis regardless of cell type. In our current study, *RASSF1* demonstrates significant differential methylation between FTC-Classic and FTC-Hurthle, with higher methylation levels in FTC-Classic, suggesting its utilization as a molecular marker to differentiate these two subtypes.

*RASSF1* encodes a signaling protein that functions through a pathway involving RAS, a component of PI3K/Akt pathway. The main mechanism of *RASSF1* inactivation appears to be through promoter methylation and loss of heterozygosity (LOH); mutational inactivation is very rare. It is thought that *RASSF1* may belong to the class of haplo-insufficient tumor suppressor genes that promotes tumor formation through the inactivation of only one allele (Schagdarsurengin, Gimm, Hoang-Vu, Dralle, & Pfeifer, 2002). Genetic alterations in genes along pathways involving *RASSF1*’s tumor suppressor role are also a consideration. Schagdarsurengin et al. (2002) examined the methylation status of *RASSF1* by methylation-specific PCR (MSP) in nine thyroid cancer cell lines and 38 primary thyroid tumors and found methylation in 70% of the FTC samples. The expression status of *RASSF1* by RT-PCR demonstrated a correlation with *RASSF1* hypermethylation and loss of transcription. Nakamura et al. (Nakamura, Carney, Jin, Kajita, & Pallares, 2005) also identified *RASSF1* methylation in thyroid cancers (PTC, FTC, medullary and anaplastic thyroid cancers, and hyalinizing trabecular tumors) and in benign follicular adenomas (FA) utilizing MSP. They found concordance between methylation of *RASSF1* and decreased protein expression in the thyroid tumors. Although 100% of their FTC samples demonstrated methylation of *RASSF1*, its methylation status was not used to differentiate the FTC subtypes as there were only 4 cases.

There have been several reports on *RASSF1* promoter hypermethylation in thyroid tumors (Xing et al., 2004; Schagdarsurengin et al., 2002) including comparisons between FTCs and their corresponding normal tissues utilizing non-quantitative methods such as MSP. Lee et al. (Lee, Geli, Larsson, Wallin, & Karimi, 2008), investigating genome-wide methylation changes utilizing Long Interspersed Nucleotide Elements-1 (LINE-1) and LUminometric methylation assay (LUMA) in 21 FTCs, observed no changes between FTCs and their corresponding normal thyroid tissues. However, *RASSF1* promoter methylation, determined by pyrosequencing and validated by MSP, was observed in 86% of their FTCs with 33% of its matched normals demonstrating methylation ≥10%. Our study is novel in that it compares the FTC subtypes, FTC-Classic and FTC-Hurthle, and their corresponding adjacent normal thyroid tissue utilizing quantitative MSP. We identified a significant difference in *RASSF1* methylation levels between FTC-Classic and FTC-Hurthle tumor tissues (p<0.001) as well as between the adjacent normal tissues (p=0.01) of these two subtypes. This suggests that *RASSF1* methylation may be used to distinguish these two FTC subtypes. When comparing *RASSF1* methylation levels between the tumor tissue and adjacent normal within an FTC subtype in the current study, as expected, the tumor tissue demonstrated higher methylation levels. This was significantly different for the FTC-Classic subtype and its adjacent normal (p<0.001), but the FTC-Hurthle subtype and its adjacent normal showed no differences in methylation levels. A pilot study (Stephen et al., 2011) by our group which looked at various thyroid lesions (normal thyroid, hyperthyroid, FTC, FTC-Hurthle and PTC) found *CASP8*, *RASSF1*and *NIS* to be frequently methylated in normal thyroid, hyperthyroid lesions, thyroid cancer and their adjacent normal. Using larger cohorts, we plan further studies to investigate the differences in methylation status between normal thyroid tissue and FTC so as to identify methylation markers that distinguish normal thyroid tissue from FTC. In our study, significant differential methylation of *RASSF1* between FTC-Classic and FTC-Hurthle tumor suggests that DNA methylation markers may be useful in discriminating among thyroid cancer subtypes.

*DAPK1*, *ESR1, NIS* and *TIMP3* also demonstrated methylation (>0.0) in our FTC samples but were not statistically significant between the Hurthle and Classic subtypes. Death-associated protein kinase 1, *DAPK1*, which is located at 9q34.1 is a positive mediator of interferon γ-induced programmed cell death in HeLa cells (Cohen, Feinstein, & Kimchi, 1997). *DAPK1* methylation frequently occurs in head and neck cancers (Worsham, Chen, Meduri, Nygren, & Errami, 2006), non-small cell lung carcinoma (Esteller, Sanchez-Cespedes, Rosell, Sidransky, & Baylin, 1999), gastric and colorectal carcinomas (Lee, Leung, Chan, Ng, & Tong, 2002; Satoh, Toyota, Itoh, Kikuchi, & Obata, 2002). In HNSCC, *DAPK1* methylation is associated with metastasis to lymph nodes and advanced stage disease (Sanchez-Cespedes, Esteller, Wu, Nawroz-Danish, & Yoo, 2000). In this study, we found an association of late stage disease with *DAPK1* methylation (p=0.034) in addition to an association with extra thyroidal extension (p=0.014) suggesting that *DAPK1* methylation maybe a marker of advanced disease and metastasis in follicular thyroid cancers.

*ESR1* is located at 6q25.1 and is involved in hormone binding, DNA binding, and activation of transcription (Ponglikitmongkol, Green, & Chambon, 1988). *ESR1* has metastasis-suppressor properties, especially in breast cancer, suggesting a tumor-suppressor role (Issa, Ottaviano, Celano, Hamilton, & Davidson, 1994). Thus, methylation mediated silencing of *ESR1* can lead to spread of a tumor. A previous study by our group on laryngeal squamous cell carcinomas identified aberrant methylation of *ESR1* as an independent predictor of late stage diagnosis (Stephen, Chen, Shah, Havard, & Kapke, 2010). In the current follicular thyroid cancer study, extra thyroidal extension (p=0.036) and late stage disease (p=0.035) are associated with *ESR1* methylation suggesting loss of its tumor-suppressor role. *ESR1* may also be involved in age-dependent increase in cancer incidence as age-dependent methylation has been detected in colon mucosa (Issa et al., 1994), cardiovascular system (Post, Goldschmidt-Clermont, Wilhide, Heldman, & Sussman, 1999), ulcerative colitis (Issa, Ahuja, Toyota, Bronner, & Brentnall, 2001), and prostate cancer. Comparisons of angiolymphatic invasion, capsular invasion and gender were also performed with respect to QMSP values, age, race, stage and extra thyroidal extension. However, they were not significantly associated with methylation of any genes or other variables.

Our long term goal is to identify methylation markers with utility in FNAs to differentiate benign thyroid, adenomas and cancer subtypes so as to determine course of management (surgery vs no surgery). Although FNA is the standard diagnostic tool for preoperative diagnosis (Segev et al., 2003), it has limitations in the presence of follicular lesions. The FTC-Hurthle variant is more easily diagnosed on FNA histology due to the presence of Hurthle cells compared to other FTC subtypes, but a definitive diagnosis of FTC, in general, requires the presence of capsular and vascular invasion which cannot be appreciated on an FNA (Ruggeri, Campenni, Baldari, Trimarchi, & Trovato, 2008). In addition, the FNA is less sensitive in detecting classic FTC and Hurthle cell carcinomas when compared to PTC (Yeh et al., 2004). Recently, a 21 gene candidate panel (inclusive of the 7 genes in this study) was evaluated for presence of methylation in matched post-surgical FNA and corresponding fresh thyroid tissue from 2 cases using QMSP. We found that 6 genes, including *RASSF1*, showed concordant presence of methylation results (Table 5). The latter suggests support for methylation markers as an adjunct to histopathology diagnoses of FNAs.

The incidence rates of thyroid cancer are almost twice as high in Caucasian Americans (CA) as in African Americans (AA) (SEER, 2014). This study was able to draw from a sizable pool of primary care patients serviced by Health Alliance Plan (HAP) and benefits from HFHS’ unique strength of a large pool of African American patients who make up about 33% of the total patient population. Of the 48 cases with self-reported race as AA and CA, over half were CA (34 vs 14), reflective of the racial incidence disparity reported for this cancer. The observed significant differential methylation of *RASSF1* (p=0.05) in AA vs CA suggests epigenetic influences, offering opportunities for further study to examine the role of DNA methylation in thyroid cancer racial disparities.

## 5. Conclusion

Together, in this study, *RASSF1, DAPK1* and *ESR1* suggest utility of methylation markers to molecularly differentiate follicular thyroid cancer subtypes for enhanced classification. Classification based on promoter methylation profiling may have preferred utility over expression profiling since such DNA-based markers are less subject to problems of tissue preservation, potential pitfalls of tissue heterogeneity, and are easily detected by PCR-based methods. Aberrant DNA methylation profiles that differentiate benign and malignant thyroid nodules may help identify potential novel diagnostic and prognostic markers, which would allow accurate early diagnosis, with personalized clinical management and surveillance. The potential reversibility of DNA methylation holds promise for these markers as potential targets for novel alternative demethylating treatments.

## Figures and Tables

**Table 1:** Cohort Characteristics

Characteristics	FTC-Hurthle (N=26)	FTC-Classic (N=27)	Total (N=53)
**Gender**			
Male	7	7	14
Female	19	20	39
**Race**			
CA	17	17	34
AA	6	8	14
Unknown	3	2	5
**Stage**			
1	9	13	22
2	9	3	12
3	6	8	14
4	1	2	3
Unknown	1	1	2

FTC - Follicular thyroid cancer; CA - Caucasian American; AA - African American.

**Table 2:** QMSP Primers and Probes

***ACTB***	
Forward Primer	TGGTGATGGAGGAGGTTTAGTAAGT
Reverse Primer	AACCAATAAAACCTACTCCTCCCTTAA
Probe	ACCACCACCCAACACACAATAACAAACACA
***CDKN2A***	
Forward Primer	TTATTAGAGGGTGGGGCGGATCGC
Reverse Primer	GACCCCGAACCGCGACCGTAA
Probe	AGTAGTATGGAGTCGGCGGCGGG
***CASP8***	
Forward Primer	TAGGGGATTCGGAGATTGCGA
Reverse Primer	AAACCGTATATCTACATTCGAAACGA
Probe	CCCGCTCCACCCTTTCCTAACACCA
***DAPK1***	
Forward Primer	GGATAGTCGGATCGAGTTAACGTC
Reverse Primer	CCCTCCCAAACGCCGA
Probe	TTCGGTAATTCGTAGCGGTAGGGTTTGG
***ESR1***	
Forward Primer	GGCGTTCGTTTTGGGATTG
Reverse Primer	GCCGACACGCGAACTCTAA
Probe	CGATAAAACCGAACGACCCGACGA
***NIS***	
Forward Primer	ATAGGGAGGTCGATACGGATATC
Reverse Primer	GAAAAAACAAAACGAAAAAAACG
Probe	TAGACGGAGCGGGGATAGGTTGTCGAGT
***RASSF1***	
Forward Primer	GCGTTGAAGTCGGGGTTC
Reverse Primer	CCCGTACTTCGCTAACTTTAAACG
Probe	ACAAACGCGAACCGAACGAAACCA
***TIMP3***	
Forward Primer	GCGTCGGAGGTTAAGGTTGTT
Reverse Primer	CTCTCCAAAATTACCGTACGCG
Probe	AACTCGCTCGCCCGCCGAA

**Table 3:** Sample Sizes, Methylation Means and Standard Deviations by Race, Stage, Extra Thyroidal Extension and FTC type

Race: AA versus CA	Variable	AA (N= 14)	CA (N= 34)	p-value
	*RASSF1*	14 (0.637 ± 0.531)	34 (0.422 ± 0.702)	0.054
	*DAPK1*	14 (0.000 ± 0.000)	34 (0.001 ± 0.003)	0.61
	*ESR1*	14 (0.000 ± 0.001)	34 (0.005 ± 0.015)	0.575
**Stage: Early vs Late**		**Early (N= 36)**	**Late (N= 15)**	
	AGE	36 (51.4 ± 13.0)	15 (66.7 ± 13.6)	<.001
	*DAPK1*	36 (0.000 ± 0.000)	15 (0.001 ± 0.005)	0.034
	*ESR1*	36 (0.000 ± 0.001)	15 (0.017 ± 0.038)	0.035
	*RASSF1*	36 (0.419 ± 0.511)	15 (0.634 ± 0.777)	0.877
**Extra Thyroidal Extension**		**No Extension (N= 41)**	**Extension Present (N= 6)**	
	*DAPK1*	41 (0.000 ± 0.000)	6 (0.003 ± 0.007)	0.014
	*ESR1*	41 (0.002 ± 0.008)	6 (0.036 ± 0.057)	0.036
	*RASSF1*	41 (0.470 ± 0.602)	6 (0.703 ± 0.693)	0.306
**FTC Hurthle vs FTC Classic**		**FTC Hurthle (N= 26)**	**FTC Classic (N= 27)**	
	*RASSF1*	26 (0.340 ± 0.751)	27 (0.730 ± 0.605)	<.001
	*DAPK1*	26 (0.001 ± 0.004)	27 (0.000 ± 0.000)	0.159
	*ESR1*	26 (0.001 ± 0.002)	27 (0.011 ± 0.030)	0.154

AA - African American; CA - Caucasian American; Early Stage - Stages 1, 2, and Unknown; Late Stage - Stages 3 and 4; FTC - Follicular Thyroid Cancer

**Table 4:** Sample Sizes, Methylation Means, Standard Deviations and Wilcoxon rank sum p-values for RASSF1 in FTC-Hurthle and FTC-Classic lesions

	FTC-Hurthle	FTC-Classic	p-value
**Tumor Tissue**	28 (0.067 ± 0.728)	29 (0.539 ± 0.596)	<0.001
**Adjacent Normal Tissue**	25 (0.022 ± 0.780)	24 (0.084 ± 0.404)	0.01
**Tumor vs Adjacent Normal p-value**	0.258	<0.001	

**Table 5:** Methylation in Matched Post-Surgical FNA and Thyroid Tissue

Genes	Thy-FNA1	Thy-T1	Thy-FNA2	Thy-T2
***NIS***	0.028122	0.02179	0	0
***RASSF1***	0.253875	0.24554	0.02155	0.03766
***TSHR***	0.095524	0.20984	0.0844	0.1315
***SERPINB5***	0.500365	0.58745	1.36001	0.59044
***SLC26A4***	0.119488	0.03552	0.00523	18.7248
***TPO***	0.595375	0.65587	1.09508	0.89846

FNA – fine needle aspiration; T – matched fresh thyroid tumor.
